# Exploring the perioperative infection control practices & incidence of surgical site infections in rural India

**DOI:** 10.1186/s13756-023-01258-4

**Published:** 2023-07-08

**Authors:** Anveshi Nayan, Bhakti Sarang, Monty Khajanchi, Nobhojit Roy, Gnanaraj Jesudian, Nandakumar Menon, Mulki Patil, Raman Kataria, Ravikumar Manoharan, Rajesh Tongaonkar, Ya Dev, Anita Gadgil

**Affiliations:** 1 Seth GS Medical College and KEM Hospital, Mumbai, India; 2 Department of Surgery, Terna Medical College & Hospital, New Mumbai, India; 3 WHO Collaboration Centre for Research in Surgical Care Delivery in LMICs, Mumbai, India; 4 Department of Surgery, Seth GS Medical College and KEM Hospital, Mumbai, India; 5 Dept of Global Public Health, Karolinska Institute, Stockholm, Sweden; 6 Association of Rural Surgeons of India, Chennai, India; 7 International Federation of Rural Surgeons, Tiruchirappalli, India; 8 Department of Surgery, ASHWINI Gudalur Adivasi Hospital, Gudalur, Nilgiris, Tamil Nadu India; 9 Department of Surgery, Karnataka Institute of Medical Sciences, Hubli, India; 10 Department of Surgery, Jan Swasthya Sahyog, Bilaspur, Chattisgarh India; 11 Department of Surgery, Tribal Health Initiative, Sittilingi, Tamilnadu India; 12 Department of Surgery, Dr Tongaonkar Hospital, Dondaicha, Dhule India; 13 Department of Surgery, Government Medical College, Kollam, Kerala India; 14 Department of Surgery, BARC Hospital, Mumbai, India

**Keywords:** Surgical Site infection, Rural hospitals, SSIs, Low and middle-income countries, Developing countries, Perioperative SSI prevention practices.

## Abstract

**Background:**

Surgical site infections (SSIs) affect around a third of patients undergoing surgeries worldwide, annually. It is heterogeneously distributed with a higher burden in low and middle-income countries. Although rural and semi-urban hospitals cater to 60–70% of the Indian population, scarce data regarding SSI rates are available from such hospitals. The study aimed to determine the prevalent SSI prevention practices and existing SSI rates in the smaller rural and semi-urban hospitals in India.

**Methods:**

This is a prospective study performed in two phases involving surgeons and their hospitals from Indian rural and semi-urban regions. In the first phase, a questionnaire was administered to surgeons enquiring into the perioperative SSI prevention practices and five interested hospitals were recruited for phase two which documented the rate of SSIs and factors affecting them.

**Results:**

There was full compliance towards appropriate perioperative sterilisation practices and postoperative mop count practice at the represented hospitals. But prophylactic antimicrobials were continued in the postoperative period in more than 80% of the hospitals. The second phase of our study documented an overall SSI rate of 7.0%. The SSI rates were influenced by the surgical wound class with dirty wounds recording six times higher rate of infection than clean cases.

**Conclusions:**

SSI prevention practices and protocols were in place in all the less-resourced hospitals surveyed. The SSI rates are comparable or lower than other LMIC settings. However, this is accompanied by poor implementation of the antimicrobial stewardship guidelines.

## Background

Surgical site infections (SSIs) affect around a third of patients undergoing surgeries worldwide, annually. SSIs are associated with increased mortality and morbidity leading to poor quality of life, prolonged hospitalisations, reduced productivity, and increased economic burden [1]. The global SSI estimates range from 0.5% to more than 30% depending on the wound classification and between the countries [2, 3]. The SSI rates in each of the wound classes are more than double in low-and-middle-income countries (LMICs) compared to those in high income countries (HICs). [3]. Indian studies have shown SSI rates ranging from 4 to 40% which vary depending on the study setting [4].

Patient-associated factors like immune-compromised status, diabetes mellitus, obesity, and advanced age increase the risk of SSIs [5]. Perioperative factors and practices like skin antisepsis, preoperative hair removal, antimicrobial prophylaxis, preoperative skin preparation, instrument sterilisation, and preoperative hand washing are the major determinants of SSIs [6, 7].

National and international collaborative studies addressing SSIs have mainly included the tertiary hospitals and medical colleges in the metropolis cities of India [3]. Rural and semi-urban hospitals cater to 60–70% of the Indian population, yet data from rural India regarding SSI prevention practices and protocols, and SSI rates are sparse. [8–10].

Quantification of the SSI burden and appropriate reporting through surveillance after discharge of the patients is essential. Knowledge of prevalent protocols and practices is necessary to identify the gaps in the perioperative practices and implement SSI preventive measures.

## Methods

### Aim

This study was initiated by the WHO Collaboration Centre (WHOCC) for surgical care delivery in LMICs, Mumbai, India, in collaboration with the Association of Rural Surgeons of India (ARSI) with the aim to describe the prevalent perioperative SSI prevention practices in rural and semi-urban hospitals and document the rates of SSIs in these hospitals.

### Study Design

**Fig. 1:**
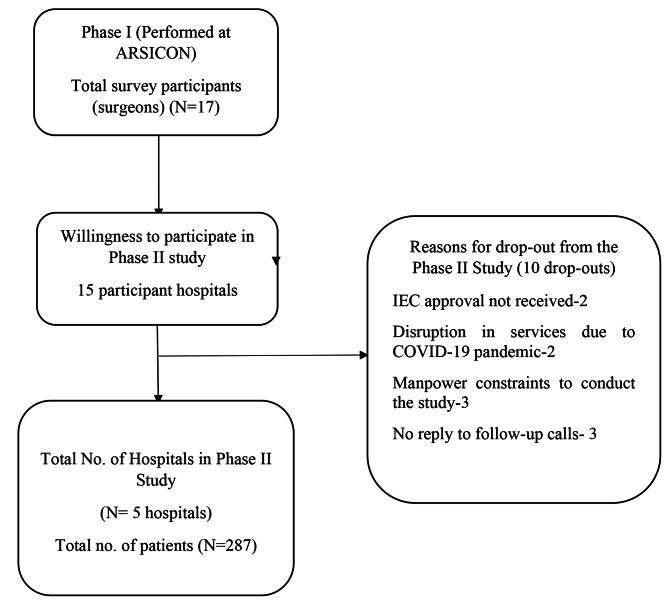
Recruitment algorithm

This was a prospective observational study performed in two phases. First phase was a cross-sectional study which consisted of administering a questionnaire to enquire into the perioperative SSI prevention practices and recruiting the hospitals for phase two. The second phase was conducted as a prospective cohort study aimed at documenting the rate and the factors affecting SSIs in the participant hospitals **(**Fig. 1**)**.

### Phase I

We conducted the first phase in November 2019 at the ‘Association of Rural Surgeons of India (ARSI)’ annual conference, which is an annual gathering of surgeons practicing in the rural and semi-urban areas of India. We assessed the infection control (SSI prevention) practices of the participant hospitals through a pre-designed questionnaire. The questionnaire was based on the World Health Organisation (WHO) Surgical Practices Guidelines, 2018 with few modifications to suit the Indian setting [11]. Before distributing the questionnaire, we made a short presentation at the gathering, explaining the importance and rationale of the study and the components of the questionnaire. We also explained the study design, aims and outcome measures of the second phase of the proposed study. Also, we approached the rural surgeons individually and explained about the various particulars of the study along with troubleshooting for any queries that they had. Only surgeons representing hospitals based in rural and semi-urban areas in India were included. Rural areas were defined as the geographical regions with population less than 10,000 while semi-urban areas were defined as the geographical regions catering to a population ranging from above 10,000 up to 1,00,000 [12]. We excluded surgeons practicing in urban areas, from this study. We did not provide any monetary incentives for participation in this study.

The questionnaire included details of the geographical location of the hospitals represented by these surgeons, a brief facility assessment, and the surgical patient load. The focus of the questionnaire was on the perioperative SSI prevention practices: like appropriate skin preparation (both surgeon’s hands and surgical site), hand washing before surgery, maintenance of the sterile surgical field, confirmation of instrument sterility, appropriate antimicrobial prophylaxis, and timing of administration of antimicrobials, complete gauze/swab counts (mop counts) after surgeries, and the use of surgical safety checklist [13]. We added a few parameters like the presence of in-house microbiology facility, availability of running water in the operation theatres (OTs) and usage of prophylactic antimicrobials beyond the OT, to suit the Indian setting based on the literature reviewed [14, 15]. We included a question to indicate willingness to participate in the second phase of the study in the questionnaire. This questionnaire-based survey was completed by the surgeons in a short time span of around 5 to 10 min. It was in English language and hence language was not a barrier for completion of the questionnaire. Adequate knowledge about duration for hand wash was defined as per WHO criteria for surgical handrub [16]. Usage of iodophors, chlorhexidine gluconate and alcohol-based scrubs was defined as ‘appropriate surgical scrub’ [16].

### Phase II

We contacted the participant rural surgeons who were willing to participate in phase II of this study and their hospitals were recruited for phase II of the study. All patients undergoing general surgical and obstetrics-gynaecological procedures needing anaesthesia, from their respective hospitals, were included in the study after due informed consent. Patients undergoing any orthopaedic procedure were excluded.

SSI was defined as infection that occurs after surgery in the part of the body where surgery took place within 30-days in the post-operative period [2]. We collected data of all consecutive operated patients over one month in the recruited hospitals. The surgeons chose the time period (one month duration of their choice) based on the convenience at their respective hospitals. Each patient at these recruited hospitals was followed up to 30 days postoperatively and evaluated for the occurrence of SSIs, hence, the study period extended up to 2 months (60 days). Prospective data collection was performed between January to March 2020 and August to October 2020. It was prolonged due to the cancellation of all elective surgical work in view of the COVID-19 pandemic from April to July 2020.

We shared audio-visual material after necessary permissions, about identification and diagnostic criteria for SSIs with the surgeons and/or representatives of the participating hospitals **[**https://globalsurg.org/ssi/index.html#/1]. Also, an educational leaflet including standard definitions for wound class and SSI diagnostic criteria were printed behind each data collection form for ready reference for the person collecting data. We had regular telephonic conversations with representatives from the participant hospitals for guidance and troubleshooting during the recruitment as well as follow-up period. The printed data collection form (proforma) included patient demographics, clinical details of the surgery, perioperative SSI prevention practices, antimicrobial usage, and timing and noting of postoperative wound checks at various intervals. SSIs diagnosed anytime during the first wound check (2nd or 3rd postoperative day), at suture removal (after 7th postoperative day) or at any point of time during the 30-day follow-up period were recorded. We also noted the antimicrobials prescribed in cases of SSIs and duration of administration of these antimicrobials.

### Data Collection

The data from each of these hospitals were collected on paper forms which were then sent to the WHOCC based in Mumbai. The data from both phases was entered on Microsoft Excel.

### Study variables

The main outcome measures for the two phases were adherence to the perioperative SSI prevention practices and the rate of SSIs at the participant hospitals, respectively. The secondary outcome measures were factors associated with SSI like age, gender, American Society of Anaesthesiologists’ (ASA) physical status class, wound class, and type of surgery (elective/ emergency).

### Statistical analysis

We performed the statistical analysis using Microsoft Excel statistical software 2019. The first phase of the study is the survey phase of the Rural SSI prevention practices and the second phase is to determine the actual SSI rates in the rural hospitals. We have done a univariate analysis in the second phase of the study to determine the effect of variables age groups, gender, ASA grades, wound class, and type of surgery on the SSI rates. The chi-square test was performed to determine the statistical significance of any associations between categorical variables and a p value of ≤ 0.05 was considered as statistically significant.

### Ethical clearance

The Ethics approval for the study was provided by ARSI through Martin Luther Christian University Research Ethics Committee.

## Results

### Phase I

A total of 17 surgeons participated in the survey. Table 1 describes the characteristics of the hospitals represented by these participant surgeons. 70% (12/17) of the participants were secondary healthcare providers. All the hospitals had running water, hand wash, and surgical scrub solutions, and sterilised instruments were available in all the hospitals.

**Table 1:** Characteristics of the survey participant surgeons/ hospitals

Variables	Count of the participating centres
**Geographic area**	
Rural Semiurban	11 6
**Level of Hospital**	
Primary Secondary Tertiary	1 12 4
**Number of Beds**	
1-100 100–500 500–1000 1000–1200	10 5 0 2
**Approximate Annual surgeries**	
< 500 500–1000 1000–5000 > 5000	8 5 3 1

Table 2 describes the perioperative SSI prevention practices of the participant hospitals, as mentioned by the surgeons in the survey. More than 76.5% (13/17) of the participants had knowledge about the WHO Surgical safety checklist although it was used partially or completely only in 35% (6/17) of the hospitals. The reasons cited by the participant surgeons for not using the WHO Checklist were time and manpower constraints. 64.7% (11/17) of the participants washed hands for an adequate duration. The prophylactic antimicrobial administered was used beyond the OTs in 82% (14/17) of the hospitals which goes against Global Guidelines for Prevention of Surgical Site Infection [16].

**Table 2:** Perioperative SSI prevention practices of the survey participant surgeons and their hospitals

	Perioperative Infection Control Practices	Number of Participants following the Standard Practice (%) (Total number of responses for each practice = 17)
1.	Availability & usage of running water	17 (100)
2.	Post-operative mop count before wound closure	17 (100)
3.	Appropriate* instrument sterilisation practices	17 (100)
4.	Appropriate* surgical scrub usage	17 (100)
5.	Non-usage of prophylactic antibiotics beyond the operation theatre (OT)	3 (17.6)
6.	Knowledge about adequate duration of hand-wash	11 (64.7)
7.	Usage of surgical safety check-list	6 (35.2)

Note: * List of standard sterilisation practices and chemicals and techniques used for scrubbing was added in the questionnaire to evaluate the appropriateness

SSI- Surgical Site Infection

### Phase II

Five hospitals participated in the second phase of the study **(**Fig. 1**)**. Of the 287 surgeries performed, 20 (7.0%) patients developed SSIs.

Preoperative shaving was done in 269 (93.7%) cases while clipping was done in 6 (2.1%) cases. 230 (80.1%) patients had taken a preoperative scrub bath. As already documented from the phase I questionnaire, the prophylactic antimicrobial prescribed preoperatively was continued for a prolonged duration in the postoperative period in 229 (79.8%) cases. In cases diagnosed with SSIs, higher classes of antimicrobials were prescribed without any definitive rationale for therapy. These prescriptions were not based on the antimicrobial culture-sensitivity reports from the wound swabs.

Table 3 describes the association of perioperative and patient factors with the occurrence of SSIs. The SSI rates were seen to be influenced by the surgical wound class with dirty wounds recording 6 times higher rates of infection than clean ones. A statistically significant increase in infection rates was observed as we go from clean to clean-contaminated to contaminated to dirty wound class The incidence of SSI was found to increase with the increase in the age of the patient. It was higher in males as compared with females. The SSI rates in patients increased with increasing ASA class. Infection rates were double in surgeries done on an emergency basis when compared with those done on elective basis.

**Table 3:** Distribution of SSI rates in patients recruited, across different variables

Variables	Operated cases	Infected cases	SSI rate (%)	P value
**Age distribution (in years)**				
0–14 15–24 25–44 45–64 > 65	18 68 97 79 25	1 4 6 6 3	5.6% 5.9% 6.2% 7.6% 12.0%	0.8
**Gender**				
Male Female	136 151	12 8	8.8% 5.3%	0.3
**ASA grade**				
I II III IV Not mentioned	154 113 10 4 6	12 7 0 1 0	7.8% 6.2% 0% 25% 0	0.6
**Wound class**				
Clean Clean-contaminated Contaminated Dirty	121 142 14 10	6 9 2 3	5.0% 6.3% 14.3% 30.0%	≤ 0.05
**Type of surgery**				
Elective Emergency	205 82	11 9	5.4% 11.0%	0.1

Notes: ASA grade- American Society of Anaesthesiologists’ physical status class

## Discussion

We documented a 100% compliance towards appropriate sterilisation practices and postoperative mop count practice at the represented hospitals, as mentioned by the surgeons, in Phase I of this study. However, the awareness about the appropriate hand-washing time and utilisation of the WHO safety checklist was limited. The prophylactic antimicrobials were continued in the postoperative period in more than 80% of the hospitals. In Phase II, the overall SSI rate was found to be 7.0%, which increased as we moved from clean to clean-contaminated to dirty.

The WHO Surgical safety checklist was used partially or completely only in 35% of the hospitals although more than 75% of the participants had knowledge about it. Time and manpower constraints were the reasons cited for not utilising the checklist. Indian urban hospital studies have shown heterogeneity in compliance rates to safety checklist from 16 to 84% [17, 18].

Among perioperative SSI prevention practices, our study showed that only about 65% of the participants had knowledge about the hand-washing time. This is comparable to the 60% that was reported by Biswas and Chatterjee in a study conducted in a tertiary care hospital in Kolkata [19].

In this study, in 79.8% cases perioperative antimicrobials continued beyond the recommended period which goes against the infection control guidelines [2]. Previously done Indian studies, too, indicate a poor compliance with antimicrobial prophylaxis guidelines. The compliance has been reported to be as low as 0% in a study in rural Madhya Pradesh to 3.9% in a study from rural Kerala [14, 15]. This is way below the adherence rates in well-resourced settings. Most studies conducted in HICs have also shown it to be below 50% [20, 21]. Although the rural surgeons scored on most of the SSI prevention parameters, poor compliance towards the appropriate usage of antimicrobials, including the timing of prophylactic administration of antimicrobials, may be a deterrent towards SSI prevention. This study highlights the need for reforms in antimicrobial prescription practices with standardised and audited protocols in these settings. Inappropriate usage of antimicrobials is associated with the emergence of resistant microbial strains without any added clinical benefits. Simple infection prevention measures with adherence to the antimicrobial stewardship guidelines has demonstrated effective infection control in an Indian urban study [22]. In 2018, the Indian Council of Medical Research (ICMR) formulated the Antimicrobial Stewardship Programme (AMSP) guidelines to curb the growing concern of antimicrobial resistance in India [23]. However, for its successful implementation in these rural settings, an evaluation of the factors driving antimicrobial prescription practices is essential. Also, in order to achieve sustainability of the SSI preventive measures, a health system strengthening approach with the provision of adequate resources, manpower, appropriate training, regular evaluations, reminders, and a culture change in accordance with the local norms may be imperative [24].

In these less-resourced rural and semi-urban hospitals, we documented an SSI rate of 7.0%. This SSI rate is comparable or only slightly higher than the overall SSI rates from HICs [3]. Also, these infection rates are lower than studies performed in other rural and semiurban LMIC hospitals in south and south-east Asian regions and comparable to ones in the urban settings [3, 22, 25, 26]. A few studies performed in tertiary care hospitals in rural India also reveal SSI rates well above 15% [10, 27]. The results from all these studies from rural tertiary care hospitals reflect the large magnitude of the SSI burden from rural India. In comparison, our study has a low SSI rate despite most participant hospitals functioning in remote areas under challenging situations. SSIs being one of the quality indicators of surgical care, these low SSI rates suggest remarkable surgical care imparted at these hospitals [28]. Further improvement of the quality of care can be ushered by addressing the gaps and implementing standard, guideline-based infection control measures in these hospitals and periodic re-assessment [13]. Instillment of good antimicrobial stewardship with continuous reinforcement of knowledge of SSI prevention, diagnosis, and treatment is very essential, even though it may take a while for favourable outcomes to be evident.

Our study showed SSI rates of 5% and 6.3% for clean and clean-contaminated cases respectively and infections were highest in the dirty operated cases. This is in accordance with literature, wherein incremental SSI rates are observed from clean to dirty cases [29]. The surgical wound class demonstrated good utility in predicting and risk-stratification of SSIs in this study. The SSI rates increased with the patient’s age, higher SSIs in male patients, but these could not be substantiated statistically. Results from other studies show a higher incidence in females and that patients more than 50 years of age had twice the risk of developing SSI when compared to those who are younger [9]. Our study registered that SSI rates for emergency surgeries were more than double that in elective surgeries, which is almost like results in other studies [9]. A risk-adjusted analysis with a larger sample size may help develop a definite association between these variables and the SSI rates.

Strengths: This study highlights the burden of SSIs from the unexplored rural and semi-urban hospitals in India. It helped in capacity building in formal research methodology since most of these hospitals were meant primarily for delivery of surgical care and were research-naive. These hospitals establish a baseline status of antimicrobial stewardship in the Indian hospitals. This study provides a comprehensive assessment of the SSI prevention practices in these resource-constrained settings. It encompasses all the six broad principles of SSI prevention. The SSI rates are documented across each of the wound classes defined by the CDC 1999 guidelines [30]. The patients enrolled were effectively followed up over a period of one-month post-surgery.

Limitations: This study included hospitals owned by the surgeons attending the ARSICON and those beyond; were not reached, which may involve a selection bias. The willingness of a surgeon to participate in a study regarding surgical site infection may reflect their eagerness to follow SSI prevention practices. The results may not be generalisable across the country as it was a mixed bag of hospitals with variations in hospital characteristics. Due to the small sample size in both phases of study and insufficient data on comorbidities, the effect of different factors on SSI occurrence cannot be commented upon statistically. Also, as the WHO checklist was not followed in most of these hospitals, compliance with each checklist factor could not be measured.

## Conclusions

Although a self-reported 100% compliance was observed towards appropriate sterilisation practices and postoperative mop count practice at the represented hospitals in this study, the awareness about the appropriate hand-washing time and utilisation of the WHO safety checklist was limited. With the existing SSI prevention practices, the SSI rates in rural and semi-urban hospitals (7%) in our study were comparable or lower than other LMIC settings. However, poor implementation of the antimicrobial stewardship guidelines has been reported. The surgical wound class demonstrated good utility in predicting and risk-stratification of SSIs.

## Data Availability

Data can be accessed with due permission of the corresponding, if required.
